# The effect of cognitive remediation in individuals at ultra-high risk for psychosis: a systematic review

**DOI:** 10.1038/s41537-017-0021-9

**Published:** 2017-05-08

**Authors:** Louise Birkedal Glenthøj, Carsten Hjorthøj, Tina Dam Kristensen, Charlie Andrew Davidson, Merete Nordentoft

**Affiliations:** 1 Mental Health Centre Copenhagen, Copenhagen University Hospital, Hellerup, DK-2900 Denmark; 2 Centre for Clinical Intervention and Neuropsychiatric Schizophrenia Research, CINS, Glostrup, DK-2600 Denmark; 3 Department of Psychiatry, Yale University School of Medicine, New Haven, CT USA

## Abstract

Cognitive deficits are prominent features of the ultra-high risk state for psychosis that are known to impact functioning and course of illness. Cognitive remediation appears to be the most promising treatment approach to alleviate the cognitive deficits, which may translate into functional improvements. This study systematically reviewed the evidence on the effectiveness of cognitive remediation in the ultra-high risk population. The electronic databases MEDLINE, PsycINFO, and Embase were searched using keywords related to cognitive remediation and the UHR state. Studies were included if they were peer-reviewed, written in English, and included a population meeting standardized ultra-high risk criteria. Six original research articles were identified. All the studies provided computerized, bottom-up-based cognitive remediation, predominantly targeting neurocognitive function. Four out of five studies that reported a cognitive outcome found cognitive remediation to improve cognition in the domains of verbal memory, attention, and processing speed. Two out of four studies that reported on functional outcome found cognitive remediation to improve the functional outcome in the domains of social functioning and social adjustment. Zero out of the five studies that reported such an outcome found cognitive remediation to affect the magnitude of clinical symptoms. Research on the effect of cognitive remediation in the ultra-high risk state is still scarce. The current state of evidence indicates an effect of cognitive remediation on cognition and functioning in ultra-high risk individuals. More research on cognitive remediation in ultra-high risk is needed, notably in large-scale trials assessing the effect of neurocognitive and/or social cognitive remediation on multiple outcomes.

## Introduction

During the last two decades there have been a surge of studies into the putative prodromal phase of psychosis commonly termed “the ultra-high risk state for psychosis” (UHR) or “the clinical high risk state”. This way of prospectively identifying individuals at heightened risk for psychosis serves as the foundation for intervention studies aimed at avoiding, ameliorating, or delaying progression to psychosis. Furthermore, initiating appropriate treatment as early as possible has the potential of improving both the clinical and functional heterogeneous outcome^1^ of UHR individuals.^2^

Cognitive deficits are prominent features of the UHR state that have received increased attention in the research field. The most recent meta-analysis on the subject found an overall impairment in neurocognition compared with healthy controls to have an effect size of Hedges’ *g* = −0.34, 95% CI: −0.43 to −0.26, with the greatest impairments found in the domains of visual and verbal memory.^3^ Moreover, evidence indicates that neurocognitive functioning can be predictive of transition to psychosis, as poorer neurocognitive functioning in the domains of verbal fluency, verbal and visual memory, and working memory have been found to be characteristic of those that develop psychosis compared with those that do not.^4–10^ Social cognitive deficits have also been identified in UHR individuals with meta-analytical evidence of a moderate overall effect size (Cohen’s *d* = −0.52, 95% Cl = −0.38 to −0.65).^11^ Studies investigating the relationship between social cognitive deficits and conversion to psychosis report mixed results, with some finding deficits in theory of mind and deficits in affect recognition/discrimination being predictive of conversion to psychosis in UHR samples,^12, 13^ while others do not find social cognitive deficits to be predictive of psychosis development.^14–20^ The magnitude of the neurocognitive and social cognitive deficits have been found to be intermediate between that of healthy controls and patients with established psychosis.^3, 11, 21, 22^

Neurocognitive deficits have a significant impact on UHR individuals’ level of functioning. In cross-sectional studies the association between neurocognitive deficits and poor role and social functioning in UHR has been identified in the areas of verbal learning and memory,^23, 24^ working memory,^25^ processing speed,^26, 27^ reasoning and problem solving,^23, 27^ and global neurocognition.^28^ In longitudinal studies, poor functional outcome has been linked to deficits in processing speed,^29, 30^ verbal learning and memory,^24, 29^ executive function and disorganized symptoms,^31^ and global neurocognitive performance.^27^ Although far fewer studies have been conducted on the association between social cognition and functioning, early evidence indicate that aspects of social cognition, such as theory of mind,^28, 32, 33^ emotion recognition,^33, 34^ and attributional bias^33^ are associated with poor functioning cross-sectionally.

As the abovementioned evidence indicates, cognitive deficits place significant impact on the course of illness and functional outcome of UHR individuals, which parallels findings from patients with established psychosis.^35–37^ Consequently, it seems essential to search for treatments that may alleviate the cognitive deficits and improve the functional outcome of UHR individuals. Cognitive remediation is a promising treatment approach aimed at reducing cognitive deficits and improving functioning in the UHR state. Cognitive remediation can be defined as “a behavioral training based intervention that aims at improving cognitive processes (attention, memory, executive function, social cognition or metacognition) with the goal of durability and generalization”.^38^ There is abundant evidence for the effectiveness of cognitive remediation in patients with schizophrenia. The most recent meta-analysis on the subject demonstrates moderate effect sizes of cognitive remediation on both global cognition (effect size 0.45, 95% CI = 0.31–0.59) and functioning (effect size 0.42, 95% CI = 0.22–0.62) at post-treatment, along with a small effect size on symptomatology (effect size 0.18, 95 CI = 0.03–0.32). Moreover, the effect of cognitive remediation on global cognition and functioning appears to be durable, as the follow-up analyses revealed effect sizes for global cognition to be 0.43, 95% CI = 0.18–0.67, and the effect size for functioning to be 0.37, 95% CI = 0.11 to 0.64, albeit no significant effect on symptoms could be found at follow-up.^38^ In contrast to numerous reviews and meta-analyses on the effectiveness of cognitive remediation in patients with established psychosis,^39–44^ no systematic review has yet been conducted assessing the effect of cognitive remediation on cognition, functioning, symptomatology, and psychosis prevention in the UHR state. Knowing that the cognitive deficits are already evident in the UHR state for psychosis, it can be hypothesized that the cognitive deficits may be more amenable to treatment at this early stage of illness, with the potential of greater brain plasticity,^45^ than at more chronic stages. Additionally, cognitive remediation interventions are effective for adolescents and young adults generally,^46^ and evidence indicate changes in cognition to be related to functional improvements in UHR individuals.^30^ Consequently, targeting cognitive dysfunctions in the UHR state for psychosis may be the optimal time to intervene when aiming at improving cognition, functioning, and quality of life of the UHR individuals.

The current study aimed at reviewing the evidence for the effectiveness of cognitive remediation on cognition, functional outcome, clinical symptoms, and psychosis prevention in the UHR population.

## Results

**Fig. 1:**
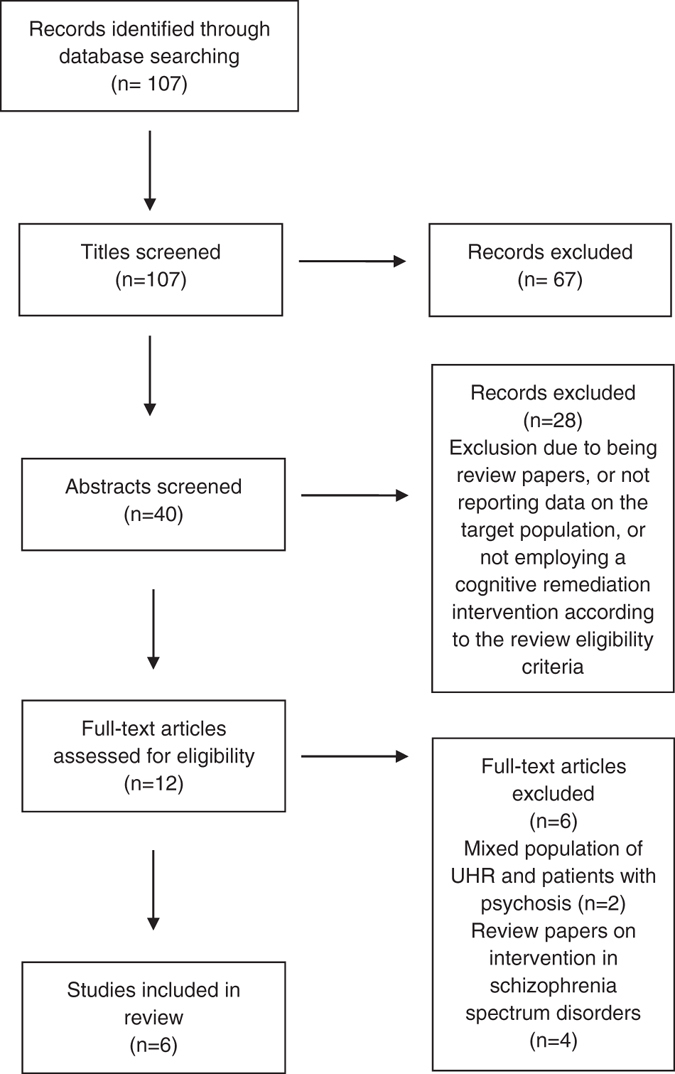
PRISMA flow diagram

The literature search resulted in 107 original articles. Out of these, six met the eligibility criteria. Figure 1 illustrates the study selection and exclusion process.

All of the six studies offered cognitive remediation within a restorative approach^47^ as opposed to a compensatory approach,^48^ and used computer-based drill-and-practice training programs. This treatment approach can also be conceptualized within a bottom-up based training framework, in which the cognitive remediation targets lower level cognitive processes,^49^ opposed to a top-down-based training approach that emphasizes training of higher order cognitive functions (e.g., problem solving and complex memory strategies).^50, 51^

One study^52^ offered cognitive remediation as part of a broad integrated treatment program, and another study^53^ offered a combined neurocognitive and social cognitive remediation program, while the last four studies offered neurocognitive remediation exclusively. In two of the trials,^54, 55^ the cognitive remediation consisted of targeted auditory training used in the Brain Fitness program (http://www.brainhq.com). The computer exercises employed in the studies were designed to improve speed and accuracy of auditory information processing while engaging auditory and verbal working memory. Two other studies^52, 56^ used the computerized digital training software from the Cogpack training program (http://www.markersoftware.com), which comprises training of the cognitive domains of attention, memory, and executive function. One study specifically targeted processing speed using the Processing Speed Training (PST) program developed by Jimmy Choi.^57^ This tablet-based, targeted training of processing speed ability intends at strengthening or resuscitating neuroanatomical connections linked to processing speed. Moreover, the intervention comprised pupillometric neurofeedback techniques that were employed to enhance and adjust the cognitive training by giving immediate biofeedback to the training software enabling it to automatically adjust the training exercises. The PST program draws on motivational theories of learning, and aims at promoting intrinsic motivation when doing cognitive remediation. Finally, the study employing both neurocognitive and social cognitive remediation^53^ used computer exercises from the Lumosity program (http://www.lumosity.com) that targeted processing speed, memory, attention, flexibility, and problem solving. The social cognitive training was done according to the SocialVille (http://www.positscience.com) training program addressing key social cognitive deficits such as social perception, emotion recognition, and theory of mind. The cognitive remediation in the studies relied predominantly on participants doing individual cognitive training, and did not include specific strategy training, or targeted training elements aiming at transferring the effect of the group training to the participants’ daily life. Table 1 displays study details and outcomes. No studies had a low risk of bias, but the risk of bias demonstrated to be lower in the randomized controlled trials (RCTs).

**Table 1:** Cognitive remediation studies in the UHR population

Study	Country	UHR sample (*N*)	Mean age^a^	UHR criteria	Experimental condition	Study design	Effect of cognitive remediation	Risk of bias
Bechdolf et al. (2012)	Germany	*N* = 1283% attrition rate	26.0	Basic symptoms (EIPS criteria)	Integrated psychological intervention: Cognitive remediation (Cogpack), cognitive behavioral therapy, skills training, psychoeducational multi-family groups12 sessions	RCT: 63 UHR in Integrated psychological intervention65 UHR in supportive counseling	Reduced rate of conversion to psychosis at 12-month and 24-month: 12-month:3.2 vs. 16.9%, *p* = .00824-month: 6.3 vs. 20.0%, *p* = .019	Selection bias: Low Performance bias: HighDetection bias: UnclearAttrition bias: LowIntention to treat: HighReporting bias: High
Piskulic et al. (2015)	USA	*N* = 3248% attrition rate	18.61	SIPS	Neurocognitive, computerized training program (Brain Fitness)40 h of cognitive training	RCT: 18 UHR in targeted cognitive remediation14 UHR doing computer games.	Improvement in social functioning (GF:Social) (t(28) = −3.26, *p* < .05) between baseline and 9-months follow-up, ***d*** ** = 3.09**Trending improvement in speed of processing between baseline and 9-months follow-up (t(29) = −2.91, *p* = .06 and between post-intervention and 9-months follow-up (t(29) = −2.99, *p* < .05)(MCCB)No improvements in cognition or clinical symptoms	Selection bias: LowPerformance bias: HighDetection bias: LowAttrition bias: HighIntention to treat: LowReporting bias: Low
Loewy et al. (2016)	USA	*N* = 8338% attrition rate	18.25	SIPS	Neurocognitive, computerized training program (Brain Fitness)Participants asked to complete 2040 h of training	RCT:50 UHR in targeted cognitive remediation 33 UHR doing computer games.	Significant improvement in verbal memory (effect size ***d*** ** = .61**). Hopkins verbal learning testrevisedNo improvements in functional outcome or clinical symptoms	Selection bias: LowPerformance bias: LowDetection bias: LowAttrition bias: HighIntention to treat: LowReporting bias: Low
Choi et al. (2016)	USA	*N* = 6210% attrition rate	18.35	SIPS	Neurocognitive training program (PST)30 h of training	RCT:30 UHR in PST32 UHR in active control group	Post-treatment: Significant improvements in processing speed: Digit symbol coding (*p* = 0.03, ***d*** ** = 0.50**)4-month follow-up: Significant improvements in processing speed: Digit symbol coding (*p* = 0.01, ***d*** ** = 0.84**).Significant improvements on self-report social adjustment (SAS-SR) *p* = 0.01, ***d*** ** = 1.04**No improvements in clinical symptoms	Selection bias: LowPerformance bias: LowDetection bias: LowAttrition bias: LowIntention to treat: UnclearReporting bias: Low
Rauchensteiner et al. (2011)	Germany	*N* = 10Attrition rate not reported	27.2	Basic symptoms (the Revised Bonn Scale for the Assessment of Basic Symptoms)SIPS criteria of: 1. Attenuated psychotic symptoms 2. Brief limited intermittent psychotic symptoms	Neurocognitive, computerized training program (Cogpack)10 sessions	Cohort study: 10 UHR16 Patients with schizophrenia	Improvementin long-term memory function and attention at post-treatmentRey-Auditory Verbal Learning Test (German version) (VLMT) D6 improvement from 10.0813.9, *p* = 0.01), ***d*** ** = 1.23**Continuous Performance Test (CPT-IP) Shapes Improvement from 0.730.81, *p* = 0.04, ***d*** ** = 0.69**No improvement in clinical symptoms	Selection^c^Comparability^c^Outcome^b^
Hooker et al. (2014)	USA	*N* = 1217% attrition rate	21.9	SIPS	Neurocognitive and social cognitive computerized training programs (Lumosity and SocialVille)40 h of cognitive training	Cohort study:14 UHR 14 healthy controls(performing as baseline reference on cognitive tests)	Significant improvements in processing speed (*p* = .01, ***d*** ** = .63**) at post-treatmentTrending improvements in visual learning and memory (*p* = .06, *d* = .54), and global cognition (*p* = .06, *d* = .45)(MCCB)No improvements in functional outcome or clinical symptoms	Selection^c^Comparability^c^Outcome

*UHR* ultra-high risk patients, *HC* healthy controls, *SIPS* the Structured Interview for Prodromal Symptoms, *EIPS* early initial prodromal state, *RCT* randomized controlled trial, *MCCB* MATRICS consensus cognitive battery, *GF:Social* Global functioning Social Scale

Risk of bias in the RCTs have been assessed according to the Cochrane criteria, with the categories of low risk of bias, high risk of bias, or unclear risk of bias

Risk of bias in the cohort studies have been assessed according to the NOS, in which a study can achieve a maximum of four stars within the Selection category, two stars within the Comparability category, and three stars within the Outcome category

^a^ UHR patients mean age at baseline

^b^ The attrition rate is reported as the proportion of the individuals in the intervention group discontinuing treatment

^c^ ***d*** = Cohens d. Effect sizes for significant between-group or within group improvements have been highlighted

Noteworthy, the study by Bechdolf et al. (2012) only reported data on transition to psychosis, and use of antidepressants, even though the registration of the trial at clinicaltrials.gov (NCT00204087) indicate that improvements in prodromal symptoms and social adjustment were secondary outcomes of the trial. This lack of reporting of secondary outcomes may indicate a reporting bias and precludes using the study to assess the effect of the intervention on functional and symptomatological outcomes.

### Effect on cognition

Five of the six studies reported the effect of cognitive remediation on cognition. Three of them were described as pilot studies (with two cohort studies), while the last two were double-blinded RCTs. All, except one study,^54^ found a beneficial effect of cognitive remediation on cognition with improvements in the domains of attention, processing speed, and memory functions. A significant improvement in verbal memory was found at post-treatment in a double-blind RCT by Loewy et al. (2016) in the group of UHR individuals receiving targeted cognitive training compared to an active control group.^55^ Improvements in processing speed were found at post-treatment, and 4-month follow-up, in a double blind RCT by Choi et al. (2016) in the group of individuals receiving targeted cognitive remediation compared to an active control group (Choi et al. 2016). Likewise, significant improvements in processing speed at post-treatment were found in a cohort study by Hooker et al. that offered cognitive remediation to a group of UHR individuals, however, the study did not include a control group. Noteworthy, the study found the improvements in processing speed to be associated with gains in role functioning.^53^ In line with the findings in the RCT by Loewy et al. (2016), a cohort study by Rauchensteiner et al. (2011) reported improvements in long-term memory functions, along with improvements in attention, in UHR individuals relative to patients with schizophrenia undergoing the same cognitive remediation program.^56^ Contrary to these positive findings on the effect of cognitive remediation on cognition, a pilot study by Piskulic et al. (2015) did not find cognitive remediation to have a significant effect on cognition at post-treatment or at 9-month follow-up. However, the study did report a statistically non-significant tendency towards improvement in speed of processing between baseline and 9-month follow-up.^54^ Noteworthy, the only study employing social cognitive remediation in combination with neurocognitive remediation did not assess the effect of this cognitive remediation approach on social cognitive outcomes.

### Effect on functional outcome

Four of the six studies evaluated the effect of cognitive remediation on functional outcomes. Two of the studies found a beneficial effect of cognitive remediation on aspects of functioning (social functioning and self-report social adjustment). In the double-blind RCT by Choi et al. (2016), lower scores on a self-report social maladjustment scale were found in participants in the intervention group compared to the active controls at 4-month follow-up. Significant improvements in social functioning between baseline and 9-month follow-up were found in the pilot study by Piskulic et al. (2015). Loewy et al. (2016) did not find an effect of cognitive remediation on either measures of global functioning, or measures of social and role functioning in their double-blind RCT. Likewise, Hooker et al. (2014) failed to find a significant effect of cognitive remediation on social and role functioning in their cohort study.

### Effect on clinical symptoms

The effects of cognitive remediation on clinical symptoms were assessed in five out of the six studies, but none of the studies reported a beneficial effect of cognitive remediation on clinical symptoms. This lack of a significant effect was seen in regard to positive and negative symptoms,^53, 55, 56^ and disorganized and general symptoms in both double-blinded RCTs and cohort studies.^53, 55^ Lack of significant effect was seen in regard to depressive symptoms in a double-blind RCT.^57^

### Effect on preventing transition to psychosis

The only trial evaluating the effect of cognitive remediation on transition to psychosis was a large scale trial conducted by Bechdolf et al. (2012). The active intervention in the trial comprised four treatment modalities: cognitive behavioral therapy, skills training, psychoeducational multi-family groups, and cognitive remediation. The study found a beneficial effect of the integrated intervention, compared to supportive counseling, in reducing the rate of transition to psychosis at 12-month follow-up and at 24-month follow-up,^52^ but as the experimental intervention was an integrated intervention, it is not possible to evaluate the separate effect of the cognitive remediation.

## Discussion

This is the first systematic review focusing exclusively on cognitive remediation in the UHR population. As the results from the six published studies indicate, there is some evidence for the effectiveness of cognitive remediation in the UHR population as a means to enhance cognition and improve functional outcome. Two of the three RCTs assessing the specific effect of cognitive remediation in the UHR state, found the cognitive remediation to have a beneficial effect on cognition in the domains of verbal memory and processing speed. The two cohort studies conducted found a beneficial effect of cognitive remediation in the cognitive domains of memory, attention, and processing speed. Moreover, two out of three RCTs that assessed gains in functional outcome, reported improvements in functional outcome in the domains of social function and self-report social adjustment as a result of a neurocognitive remediation program. One of the studies reporting a beneficial effect of cognitive remediation on functional outcome was a methodologically rigorous, double-blind RCT demonstrating a low risk of bias,^57^ while the other was a pilot study.^54^ The RCT not finding a significant effect on outcome does suffer the methodological limitation of having a high attrition rate.^55^ No effect on functional outcome was reported in the one cohort study assessing this outcome.

None of the studies found a significant effect of cognitive remediation in regard to clinical symptoms (i.e., positive, negative, disorganized, general, or depressive symptoms). It is not possible to assess the effect of cognitive remediation on preventing transition to psychosis, since the only study addressing this issue offered cognitive remediation as a part of an integrated treatment, precluding any conclusions to be drawn regarding the separate effect of the cognitive remediation on psychosis prevention. The two largest studies conducted to date by Loewy et al. (2016) and Choi et al. (2016), which have assessed the direct effect of cognitive remediation in the UHR population, are strengthened by being RCTs with a low risk of bias. These studies both show encouraging results regarding the effect of cognitive remediation on cognition, and on functional outcome in one of the studies. However, it must be emphasized that the evidence level is still low, as the effect of cognitive remediation in the UHR population has not been thoroughly investigated at this stage. The remaining studies reviewed were pilot studies with small sample sizes, and thus they may have been statistically underpowered to detect significant effects of the cognitive remediation. Moreover, it must be emphasized that two studies were cohort studies, lacking a control group, which may raise concerns about the risk of bias. Speculating, it may be that the equivocal findings on the effectiveness of cognitive remediation on functional outcome and symptoms may be due to the assessments being carried out too early to see a strong effect in these domains, as evidence from patients with schizophrenia suggest that functional and symptomatological improvements of cognitive remediation may manifest themselves over the longer-term (e.g., 6-month follow-up).^58^ This underlines the need for long-term follow-up of cognitive remediation studies in the UHR population.

It is noteworthy, that only three of the studies; the RCT by Choi et al. (2016), the RCT by Bechdolf et al. (2012), and the cohort study by Rauschensteiner et al. (2011) delivered the cognitive remediation as a group-based training. Additionally, in the Choi et al. (2016) study, the group-based approach included a facilitator being present in the group sessions to assist participants in case of questions (e.g., on loading the remediation program, or explaining how the computer exercises work). In the remaining three studies, the cognitive remediation was done individually at the participants’ home, or at the research facility, with no or minimal support offered by a facilitator. As mentioned in the Result section, all the studies offered cognitive remediation within a bottom-up training-based framework targeting basic cognitive processes. This suggests the need for future studies investigating the effect of top-down-based approaches to cognitive remediation, which have been found to result in cognitive and functional gains in patients with schizophrenia.^59^ All the studies offered a cognitive training dose of 20–40 h, except the studies by Bechdolf et al. and Rauschensteiner et al. offering 12 and 10 h of training, respectively. It may be speculated that these delivery characteristics of the cognitive remediation may have influenced the equivocal results that was achieved, although evidence from patients with schizophrenia indicate that the differences in the delivery of the cognitive remediation do not seem to be critical to the benefit of the cognitive remediation on cognitive outcome.^38, 39^

Evidence suggests motivational deficits to be central features of schizophrenia spectrum disorders,^60, 61^ that may critically affect the ability to engage in and benefit from cognitive remediation.^62^ Hence, it may be essential to address motivational aspects directly (e.g., using motivational interviewing, motivational booster meetings, or cognitive remediation programs, such as the Neuropsychological and Educational Approach to Remediation model designed to target intrinsic motivation^51^) when doing cognitive remediation in a UHR population. Motivational aspects were addressed in the trial by Choi et al. (2016) that notably demonstrated a rather low attrition rate compared to other cognitive remediation trials in the UHR population showing high attrition rates.^54, 55^ This high attrition rate in many of the trials constitutes an important bias when interpreting the effectiveness of the cognitive remediation programs. This also points to the need for the development of new ways of delivering cognitive remediation that are engaging for the participants as this is essential for the feasibility and scalability of cognitive remediation programs in clinical practice.

Additionally, meta-analytical evidence from patients with schizophrenia indicate that an enhanced effect of cognitive remediation can be achieved when provided in the context of psychiatric rehabilitation.^38^ This suggests the need to offer cognitive remediation along with other treatment modalities to maximize the effect on outcome.^52^

Only one of the trials reviewed offered social cognitive remediation in addition to neurocognitive remediation, but did not include any social cognitive outcome measure, which precludes any conclusions to be drawn on the effect of cognitive remediation on social cognitive function. Social cognition has been found to be highly impaired in UHR individuals,^11, 21^ and social cognition may be proximal to the functioning of UHR individuals,^32–34^ and patients with schizophrenia.^63^ Evidence from patients with schizophrenia points to the beneficial effect of social cognitive remediation on both social cognitive function and functional outcome.^64^ Hence, there is a need for remediation studies targeting social cognitive function in UHR individuals. Given that both neurocognition and social cognition are impaired in UHR individuals it may be speculated that a combined treatment approach may enhance the effect on outcome. Beneficial results of such combined treatment approaches have been found in patients with schizophrenia with improvements seen in relation to both neurocognition, social cognition, functional outcome, and symptomatology.^65, 66^

### Methodological considerations

A strength of the review is that a high effort was put into identifying studies meeting the eligibility criteria by contacting numerous key researchers in the area, in addition to the systematic literature search. That did not result in any additional studies being identified, indicating that the literature search had been optimal. Moreover, the studies reviewed used related computer-based training programs, aimed at restoring cognitive function, which enhances the comparability. An additional strength is that risk of bias in the studies reviewed were assessed according to the well-defined criteria of the Newcastle–Ottawa Scale (NOS) for cohort studies, and the Cochrane review criteria for RCTs. Limitations are that only very few studies have been conducted on the effect of cognitive remediation in the UHR state, and thus the results must be interpreted with caution. Moreover, the studies differed in the criteria used to identify UHR individuals, and on demographic parameters such as age and use of medication. Furthermore, the studies used different outcome assessments on cognition, functioning, and symptomatology, precluding a meta-analysis to be conducted on these outcomes.

## Conclusion

To conclude, evidence is still scarce on the effectiveness of cognitive remediation in the UHR population, and more research is needed. The six studies published to date provide preliminary evidence for the effectiveness of cognitive remediation on cognition and aspects of functional outcome, but methodological considerations can be raised regarding the majority of studies, precluding any firm conclusions to be drawn. None of the studies reviewed deployed targeted social cognitive training, or assessed social cognitive function as an outcome. Hence, there is a need for methodologically rigorous trials that may not only confirm the abovementioned findings, but also provide additional evidence on the effectiveness of neurocognitive and/or social cognitive remediation on multiple aspects of cognitive outcome, functional outcome, clinical symptoms, along with the potential of preventing transition to psychosis. Additionally, future research is warranted into the effectiveness of cognitive remediation in conjunction with other treatment components in UHR populations (e.g., vocational rehabilitation, exercise, oxytocin) as it has been done in patients with established psychosis,^67–70^ as this treatment augmentation may enhance the effect on outcome.

Given that the UHR paradigm is proving useful in identifying help-seeking patients that may never develop a psychotic disorder, but are still troubled by functional deficits in the majority of cases,^71^ there is a need for safe and tolerable interventions in this population. This review suggests cognitive remediation to be a viable way to affect cognition and functional outcome, although many questions on its effectiveness still need to be answered.

## Method

### Study registration

The study adheres to the PRISMA (Preferred Reporting Items for Systematic Reviews and Meta-Analyses) guidelines. The protocol for the study was registered at PROSPERO international prospective register of systemic reviews (registration number CRD42016047980), before initiation of the literature search.

### Search strategy

A literature search was conducted from inception until September 2016 in the electronic databases of MEDLINE, PsycINFO and Embase using the following words: (“at risk mental state” or “UHR” or “clinical high risk”, or “prodromal”, or “prodrome”, or “at risk psychosis”) and (“cognitive remediation”, or “cognitive rehabilitation”, or “cognitive training”).

### Eligibility criteria

Articles were included if they provided cognitive remediation, or an intervention designed to enhance cognitive functioning,^39^ and used a clinical validated measure of the UHR state, i.e., the Comprehensive Assessment of At-Risk Mental States,^72^ the Structured Interview for Prodromal Symptoms (SIPS),^73^ or the basic symptoms approach [e.g., Early Initial Prodromal State (EIPS) criteria].^74, 75^ Studies were included irrespective of whether they included a control group or not. Both RCTs and cohort studies were included. While we acknowledge that it may be challenging to evaluate treatment effect in cohort studies, and that RCTs are considered the ideal way to evaluate treatment effect, we did include both RCTs and cohort studies in this review, as it intends to convey a preliminary description of the current evidence for the effectiveness of cognitive remediation in the UHR state. A primary emphasis when interpreting the results is, though, put on the results obtained from RCTs. Articles were excluded if they were in other languages than English, or did not report results separately for the UHR group. Four conference papers meeting the eligibility criteria of the review were identified. The authors of these papers were contacted to clarify if any publications on the data were underway. Moreover, numerous key researchers in the area were contacted to identify any potential, additional cognitive remediation studies approaching publication. That did not result in any additional papers being identified.

Risk of bias in the RCTs were assessed according to the Cochrane criteria,^76^ operating with the categories of low risk of bias, high risk of bias, or unclear risk of bias. Risk of bias is assessed in the areas of selection bias, performance bias, detection bias, attrition bias, and reporting bias. Selection bias is assessed based on information on biased allocation to interventions due to random sequence generation or allocation concealment. Performance and Detection bias is assessed based on information on partly blinding of participants or personnel during the study; and secondly blinding of outcome assessors. Attrition bias is evaluated based on amount, nature or handling of incomplete outcome data; and Reporting bias is concerned with the risk of selective outcome reporting. Other bias not addressed in the domain might be reported, if important concerns or specific questions might affect study quality.

Risk of bias in the cohort studies were assessed according to the NOS,^77^ which is recommended by the Cochrane Collaboration for assessing the quality of published non-randomized studies. NOS can be used as a checklist or scale, and contains eight items, categorized into three broad dimensions: the selection of study-groups, the comparability of groups; and the assessment of the outcome of interest. For each item, response options are provided. A star system is used to allow a semi-quantitative assessment of study quality, assigning up to a maximum of nine points for the least risk of bias (provided in the following manner: four stars within the Selection category, two stars within the Comparability category, and three stars within the Outcome category).
